# Enhancing TreeMMoSys with a high-precision strain gauge to measure the wind-induced response of trees down to the ground

**DOI:** 10.1016/j.ohx.2022.e00379

**Published:** 2022-11-17

**Authors:** Julius Nickl, Sven Kolbe, Dirk Schindler

**Affiliations:** Environmental Meteorology, University of Freiburg, Werthmannstrasse 10, D-79085 Freiburg, Germany

**Keywords:** Wind-tree interaction, Storm damage, Natural hazard, Environmental monitoring

## Abstract

Measuring tree response to wind loads is fundamental for the process-based analysis of wind-tree interactions. Comprehensive knowledge of wind-tree interactions enables the further development of decision support tools available for estimating the probability of wind damage to trees. The assessment of critical wind loads that lead to damage is particularly important. This paper describes the inexpensive Tree Strain Sensor (TSS) suitable for precisely measuring the response of tree parts to external loads such as pulling tests and natural wind loading. It is an addition to the recently developed Tree Motion Monitoring System (TreeMMoSys) but can also be used as a standalone device, allowing measurements necessary to estimate effective wind loads on trees.

## Graphical abstract

**Figure:**
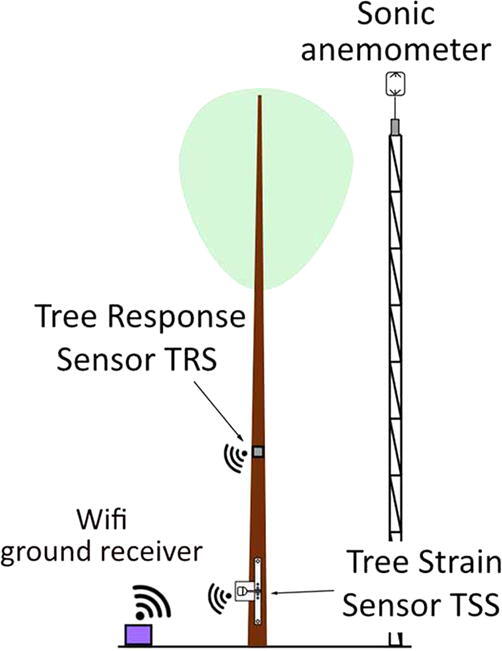

## Nomenclature

Abbreviations: Description
*f_0_*: fundamental sway frequency (Hz)
*f_1_*: frequency of the first harmonic (Hz)
*M*: above-canopy momentum flux density (m^2^/s^2^)
*MAE*: mean absolute error (µE)
MQTT: Message Queuing Telemetry Transport communication protocol
PRPT: pull-and-release test
*PSD*: Welch power spectral density
PTQ: Picus TreeQinect
RT: root
*R^2^*: coefficient of determination
ε*_x_*: stem strain in x direction (east–west)
ε*_y_*: stem strain in y direction (north–south)
ε*_RT_*: strain measured along the longitudinal root axis
*E*: stem strain vector
SPT: static tree pulling test
*T*: stem tilt vector (°)
*t_x_*: stem tilt in x direction (east–west) (°)
*t_y_*: stem tilt in y direction (north–south) (°)
TreeMMoSys: Tree Motion Monitoring System
TRS: Tree Response Sensor
TSS: Tree Strain Sensor
*u*: horizontal wind vector component in x direction (east–west) (m/s)
u′: fluctuation of *u* (m/s)
*v*: horizontal wind vector component in y direction (north–south) (m/s)
v′: fluctuation of *v* (m/s)
*w*: vertical wind vector component (m/s)
w′: fluctuation of *w* (m/s)
*z*: measurement height above ground (m)

Specification table

**Table:** 

Hardware name	Tree Response Sensor (TSS)
Subject area	Environmental, forest and agricultural sciences, educational tools
Hardware type	Strain sensor, field measurements, mechanical engineering
Open Source License	GNU General Public License v. 3
Cost of Hardware	324.26 € for an operational system with 10 sensors
Source File Repository	https://osf.io/p8h6k/

### 1 Hardware in context

Assessing wind loads and their impact on tree motion is a prerequisite for understanding wind-tree-interactions and the processes that lead to wind damage to trees and forests [1], [2]. To return to their rest position as quickly as possible and prevent damage after exposure to wind loads, trees transfer the external forces, first acting on the crown periphery through the branches and stem to the roots [3].

Essential knowledge about the response of full-scale trees or tree parts to natural wind loading comes from field studies that mostly measured local tilt [4], strain [5], [6], [7], [8], [9] or acceleration [10] of the stem, branches [11], and roots [3] at multiple positions [12]. To capture important wind load and tree response components for wind-tree-interaction analysis, the measuring devices are sampled at frequencies up to 20 Hz [11]. These sampling rates allow the measurement data analysis in the frequency domain. Previous studies show that the correlation between high frequency components wind excitation of forest trees and wind-induced tree response is primarily low, which complicates the physical interpretation of wind-induced tree motion [13].

The increasing number of field studies in which wind-induced tree response has been measured is steadily leading to improvements in the understanding of wind-tree interactions under diverse environmental conditions [14]. Previous field studies found that the motion of forest-grown trees induced by weak to moderate wind loading is dominated by sway in the fundamental mode [15], [16], [17]. As the wind load increases, coherent turbulent structures control forest tree motion more [13], [18], [19]. At the same time, the importance of sway in the fundamental mode decreases [20], [21]. Furthermore, the sway behavior of trees is related to their above-ground architecture [14], size [22], competition [23], [24], and other environmental conditions such as air temperature [25] or water shortage [26].

The motion of trees in the wind causes strain in all above- and below-ground tree parts [2], [27]. Therefore, numerous earlier studies used strain gauges for monitoring wind-induced tree motion [8], [28]. The measured strain is related to tree characteristics such as bending moment and modulus of elasticity which are essential for assessing storm damage risk [29]. They can also be used to monitor the change in dynamic characteristics associated to tree growth and long-term effects [30]. Other studies showed that strain measured at lower stem parts is related to wind speed [22] and used for predicting critical wind speed values [9], [28] required to damage forest trees [31], [32].

We describe the low-cost Tree Strain Sensor (TSS), used to measure local strain at single or multiple positions in tree parts under natural wind conditions. TSS can be used as a standalone system (any device with a Message Queuing Telemetry Transport broker) or as part of the Tree Motion Monitoring System (TreeMMoSys) [33]. It can easily be mounted to tree parts and is scalable in terms of the number of sensors. Details on the sensor assembly, recording, and processing of measured strain data are presented in this paper.

### 2 Hardware description

#### 2.1 Strain measurements

The developed sensor is based on the design presented for the first time in an earlier study [5], fully described in another study [6], and used elsewhere [7], [22]. These studies show that strain (ε) measured on tree parts, e.g., stem and branches, is linearly proportional to their displacement. This linear relationship can be exploited by means of a caliper type aluminum transducer tightly screwed to the tree parts at two points. The aluminum transducer carries four strain gauges (type Taidacent BF350, Taidacent, China) that measure its length variation induced by tree part motion. From these measurements, strain is calculated as:

(1) ε=ΔL/L

where Δ*L* is the wind-induced length variation of the aluminum transducer between the two mounting points with a distance (*L*) of 170 mm.

**Fig. 1:**
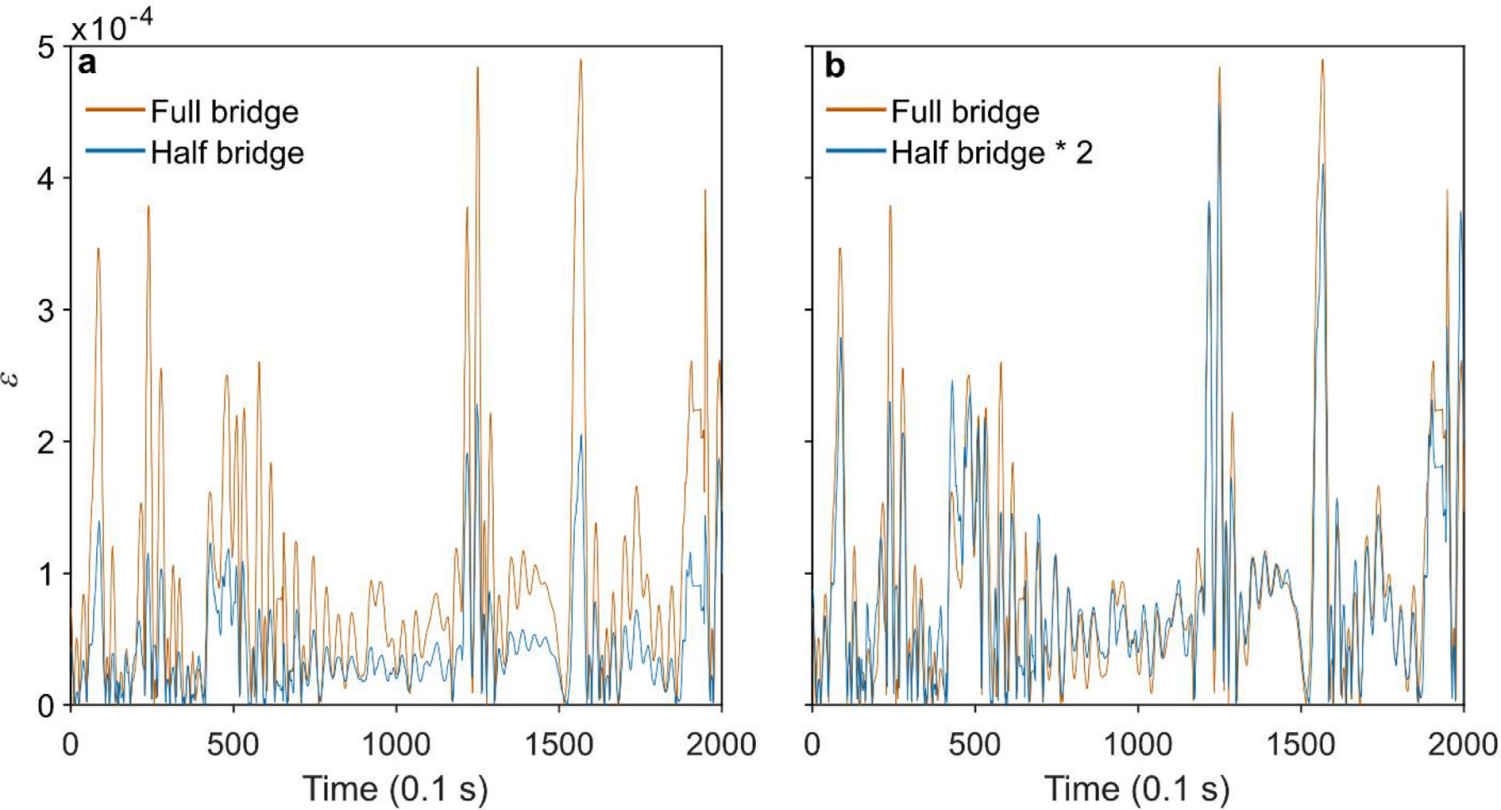
(a) Comparison of TSS signals measured with half and full Wheatstone bridge configurations over 200 s at a frequency of 10 Hz. (b) Comparison of 2 × half-bridge signal with full-bridge signal.

Since a temperature-dependent variation of the zero value of strain gauges was reported in earlier studies [5], [7], [22], we use a full Wheatstone bridge configuration with two active and two inactive strain gauges. In this configuration, the strain gauge is temperature-compensating by itself. Moreover, the full Wheatstone bridge configuration has a higher sensitivity than other Wheatstone bridge configurations [34]. We applied a TSS sampling frequency of 10 Hz for the measurement purpose presented here. The measured strain data is recorded with the TreeMMoSys ground receiver [33]. Fig. 1 compares TSS signals measured with half and full Wheatstone bridge configurations. The full-bridge configuration has higher sensitivity in measuring the applied loads [34], [35]. The amplitude of the full-bridge signal is greater by about a factor of 2 (Fig. 1a) than the half-bridge signal (Fig. 1b).

#### 2.2 Power consumption and system output

The total power consumption adds from the TreeMMoSys ground receiver (0.13 A, [33]) and the number of TSS (26.3 mA per TSS) used. Using 10 TSS, the system can be operated by a 60 Ah 12 V battery for 152 h. The system can either be used calibrated or uncalibrated. In the uncalibrated case, the system provides information about the sway characteristics of the investigated tree part in the frequency or time–frequency domain. Calibrated sensors can also provide data on wood fiber length change from micro- to millimeters.

#### 2.3 Data infrastructure

**Fig. 2:**
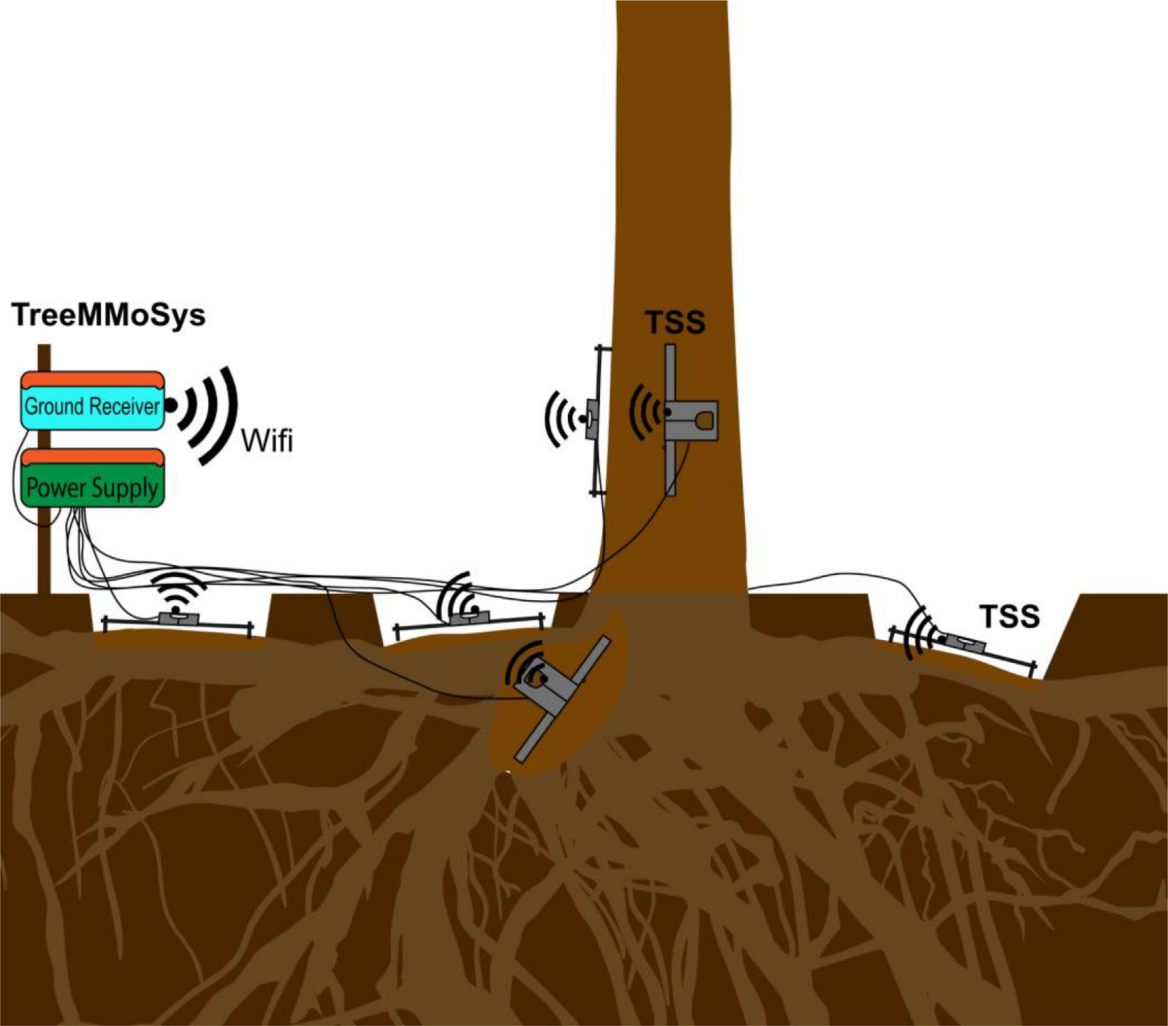
Setup of six Tree Strain Sensors (TSS) measuring strain at the stem base (*z_1_* = 0.2 m) in two directions and at the top of three main roots. The measured data are sent via Wifi to the Tree Motion Monitoring System (TreeMMoSys) ground receiver.

The voltage signals of the Wheatstone bridge are amplified by an HX711 signal amplifier. A WEMOS D1 mini microcontroller is used to read the HX711 output signals and sends them via Wifi to the TreeMMoSys ground receiver using the Message Queuing Telemetry Transport (MQTT) communication protocol (Fig. 2).

### 3 Design files

The design files for the ground receiver and the power supply were presented in an earlier study [33]. The schematic drawing of the aluminum transducer and the file to program the Wemos D1 mini is listed in Table 1.

**Table 1:** List of design files.

Design filename	File type	Open source license	Location of the file
TSS.ino	INO	GNU GPL v3	https://osf.io/khctz/
Schematic drawing TSS.tiff	TIFF	GNU GPL v3	https://osf.io/u5k74/

**Fig. 3:**
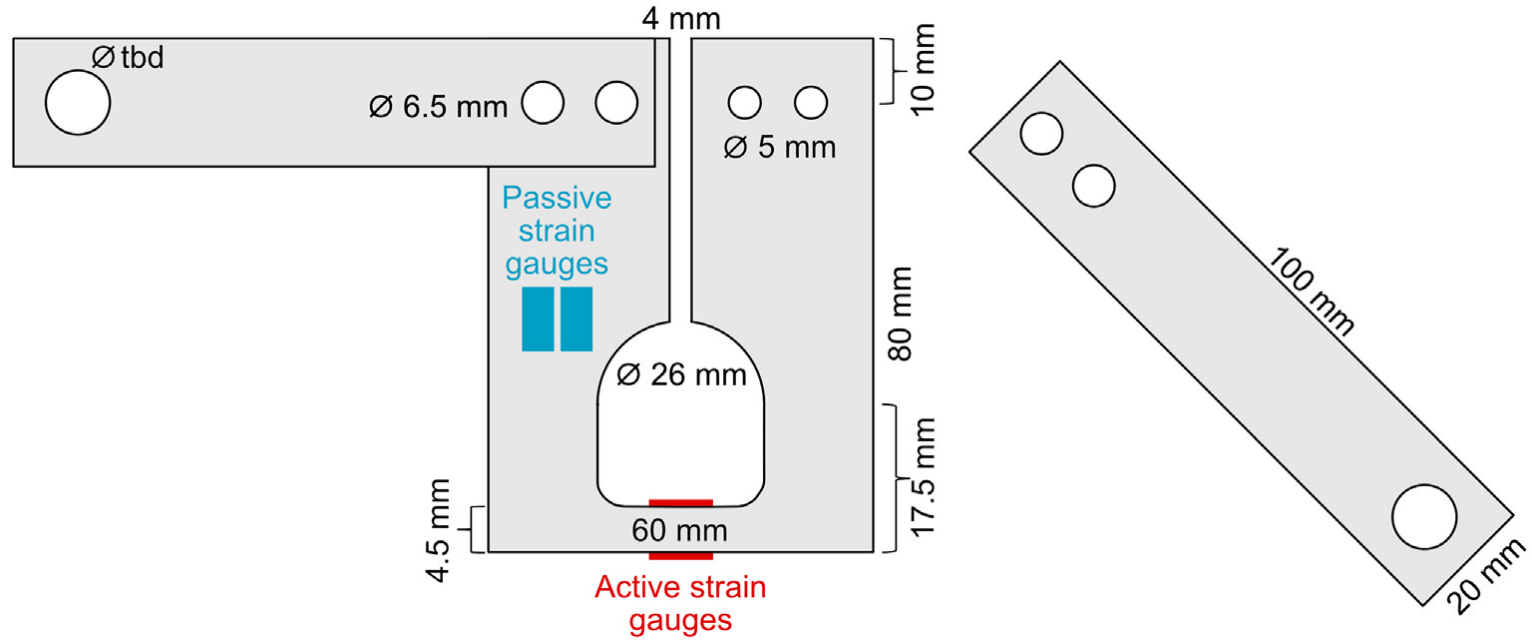
Schematic drawing of the aluminum strain transducer in a full Wheatstone bridge configuration with extension arms.

The aluminum transducer and the extension arms can be milled into shape after Fig. 3. They have similar dimensions as the strain gauge transducer described earlier [6]. In the present work, the original design was modified to allow the full Wheatstone bridge circuit assembly. The cut-open-side of the aluminum transducer is 4.0 mm. This is wide enough to avoid contact of the open ends when the transducer is mounted to the sampled tree parts and under compression. If higher compressions are expected the size of the cut-open end can be modified accordingly.

**Fig. 4:**
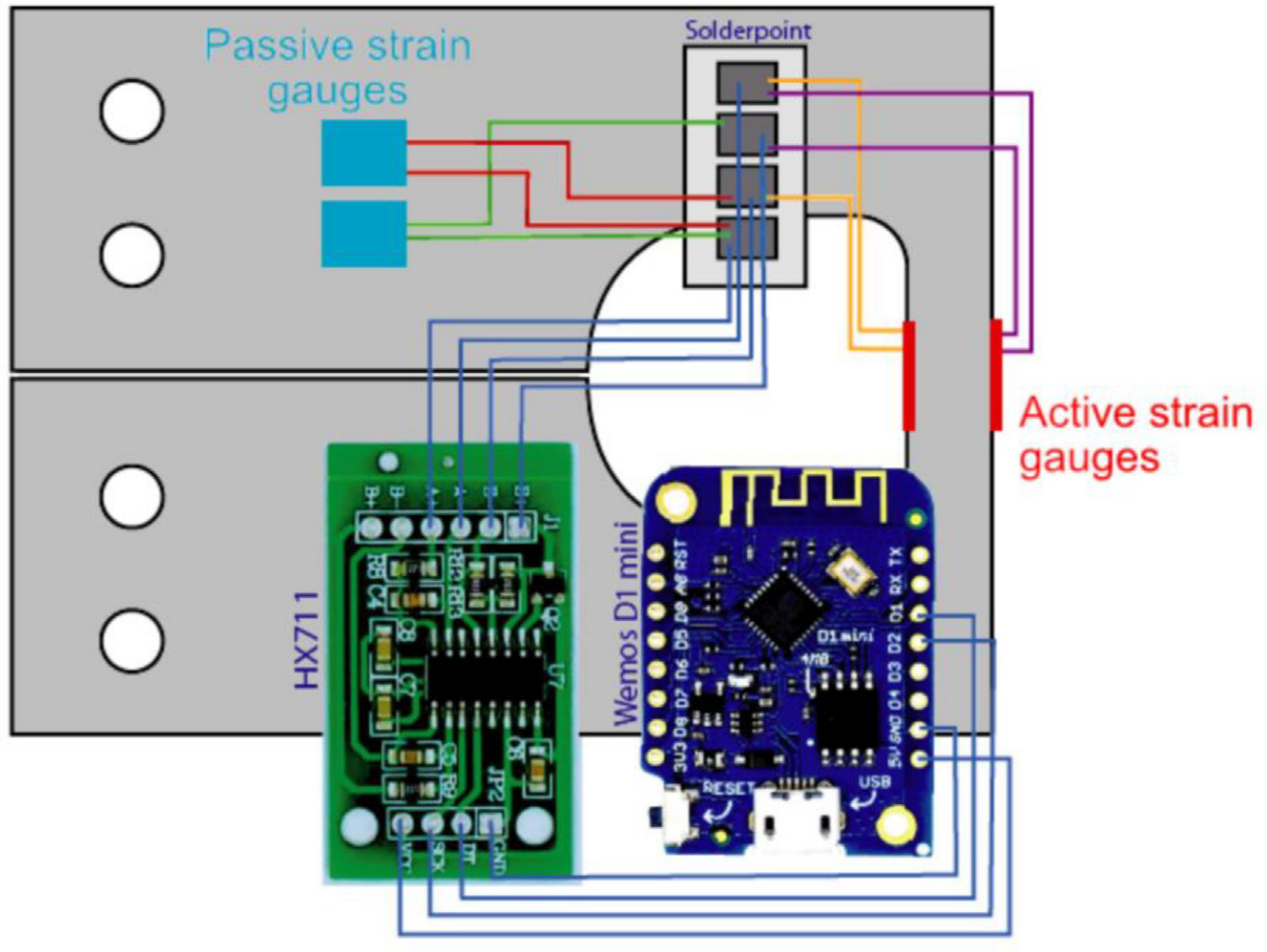
Wiring of the Tree Strain Sensor (TSS). Blue lines represent enameled wires while green, red, golden and purple lines represent the wires of the pre-soldered strain gauges, connected into a full Wheatstone bridge circuit [36], [37]. (For interpretation of the references to colour in this figure legend, the reader is referred to the web version of this article.)

The wiring of the strain gauges into a full Wheatstone bridge circuit and onto the HX711 as well as the connections to the Wemos D1 mini are presented in Fig. 4.

### 4 Bill of Materials

The Bill of Materials for the TreeMMoSys ground receiver and the power supply are described in a previous study [33]. For TSS it is given in Table 2.

**Table 2:** Bill of Materials for a TreeMMoSys ground receiver, power supply, and 10 Tree Strain Sensors (TSS). Links to online available materials are given in the appendix.

ID	Component	Quantity	Unit (EUR)	Total (EUR)
01	TreeMMoSys Ground receiver	1	52.99	52.99
02	TreeMMoSys Power supply	1	56.46	56.46
03	Milled Transducer	10	5.00	50.00
04	Cutted and Drilled Extension Arms	20	2.00	40.00
05	D1 mini - ESP8266	10	3.00	30.00
06	HX711 module	10	0.90	9.00
07	Screw M6x20mm	40	0.21	8.40
08	Woodscrew 50x5mm	20	0.80	16.00
09	3 M Scotch-Weld® SF20 Instant Adhesive	2 g	0.75	1.50
10	Enamelled wire, 0.35 mm	5 m	0.05	0.25
11	Mibenco® liquid rubber white	20 g	0.04	0.20
12	Double Sided Foam Adhesive Tape	1 m	1.30	1.30
13	Rescue Blanket	1	1.00	1.00
14	Taidacent BF350- 3AA pre-soldered strain gauges	30 (6 spare)	1.39	41.7
15	tesafilm® eco & clear tesafilm® self-adhesive tape	1	1.50	1.50
16	Hole grid board	1	0.10	0.10
17	Twin-Stranded-Wire 0.14 mm^2^	3 m	0.32	0.96
18	Micro USB Plug Type B	10	1.05	10.50
19	Conformal Coating M.G. Chemicals 419D	10 g	0.24	2.40
		**Grand total:**		**324.26 €**

### 5 Build instructions

The build instructions for the TreeMMoSys ground receiver and the power supply was presented in a previous study [33]. The build instructions for TSS are as follows:

1. See TreeMMoSys build instructions for ground receiver V2 [33].
2. See TreeMMoSys build instructions for power supply V2 [33].
3. Mill the transducer and extension arms according to Fig. 3. The hole in the extension arm for the attachment to the tree parts has to be sized accordingly to the woodscrews intended to use. For usage on the stem, we found screws with 8.0 mm diameter sufficient; for usage on roots we used 4.0 mm diameter screws.
4. The following mounting of the strain gauges requires a work bench with a small vice. Briefly sand the selected mount surfaces for the strain gauges on the transducer. Clean the surfaces with isopropanol alcohol or a comparable detergent. Mark the exact position of the strain gauge with a pencil.
5. Take a strip of adhesive tape and lay it flat on the workbench, adhesive side looking upward. Use a tweezer to carefully take a strain gauge by a corner and stick it with the upward facing side onto the adhesive tape. Place the strain gauge with the tape on the intended position and stick it on one side onto the surface (Fig. 5a). Apply a small drop of instant adhesive on the intended position of the strain gauge on the aluminum transducer. Roll the adhesive tape with the strain gauge sticking under it evenly onto the drop and the strain gauge on it in the intended position. Apply thumb pressure for roughly a minute by placing your thumb onto the tape. The warmth will help to cure the instant adhesive. Remove the adhesive tape by pulling carefully at a shallow angle (Fig. 5b).

**Fig. 5:**
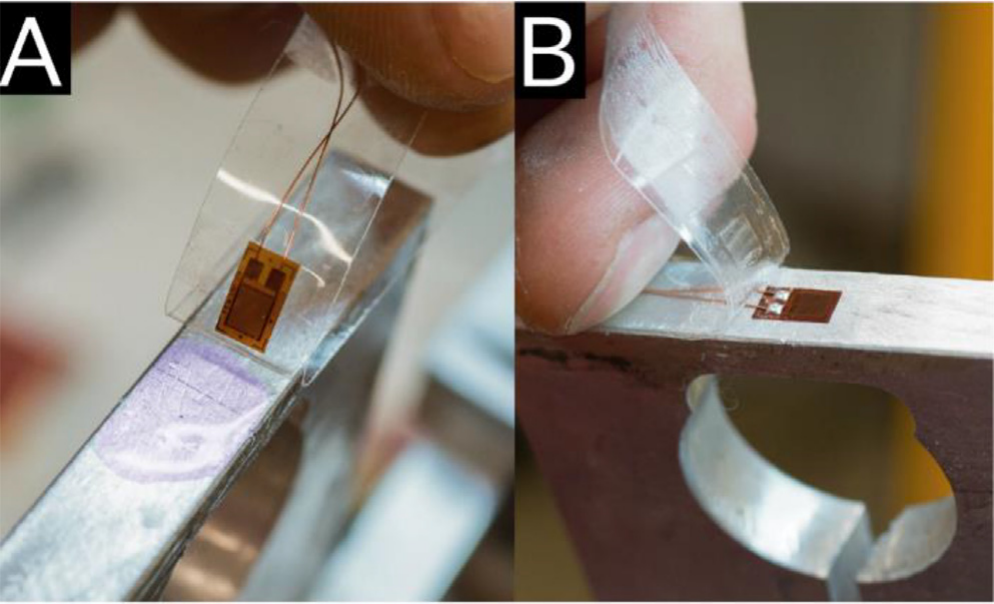
Application of a strain gauge. (a) Strain gauge glued onto the adhesive tape, with the instant adhesive (violet drop) already in place, the amount of adhesive shown here is exaggerated for better visibility. (b) Removal of the adhesive tape and the strain gauge glued onto the aluminum surface. (For interpretation of the references to colour in this figure legend, the reader is referred to the web version of this article.)
6. Apply a thin coat of liquid rubber directly onto the strain gauge to protect it from moisture. It is recommended as the glue deteriorates with exposure to humidity over time.
7. Solder the full Wheatstone bridge: Cut a hole grip board to a size of 3 × 12 holes to make the solder point. Apply a sufficient amount of solder to it and solder the wires of the strain gauge according to Fig. 4. Solder the connections between the soldering point and the corresponding pins of the HX711 board with the enameled wire, as well as the connections between the HX711 and the Wemos D1 mini.
8. Mount the soldering point, the HX711, and Wemos D1 mini with a short piece of double-sided-adhesive-foam-tape onto the aluminum transducer, covering all electric connections on the bottom site of these items. This is used to ensure an electric isolation and avoid electric shortcuts.
9. Cut the twin-stranded wire into 15 cm pieces and solder the male micro USB ports onto one end of the wires. Via these wires the system will be powered in the field, by connecting the male micro USB ports with the female micro USB ports on the WEMOS D1 mini. The cut-open end will be connected with the power supply.
10. Install the Arduino desktop application onto your PC [38]. Add the URL https://arduino.esp8266.com/stable/package_esp8266com_index.json to the “additional board managers” option, found in the app settings. Connect one WEMOS D1 mini via a micro USB cable to your PC. Load the TSS.ino script (available online: https://osf.io/p8h6k/) onto the WEMOS D1 mini, make sure to set “Sensor_ID” to a continues number and adapt - if necessary - the SSID, following the instructions for the sensor setup described in an earlier study [33]. Check the function of the sensor by looking at the serial monitor. The successful mounting of the strain gauges can be tested by applying small loads on the extension arms.
11. Trouble shooting:
  a. If the message “HX711 can’t be found” appears, check the connection and power connection between the HX711 and WEMOS D1 mini.
  b. If the sensor reading is a fixed value, the HX711 works fine, but the solder point is connected incorrectly.
12. If everything works, cover all electronics with a thin layer of conformal coating to enable the TSS deployment in the field.
13. Prior to the installation, wrap TSS in a 1 m × 1 m piece of a space blanket to provide radiation shielding.

### 6 Validation and characterization

#### 6.1 TSS calibration

For calibration, the extension arms of the TSS are attached to two adjustable wood blocks connected with a fine threaded rod. In the rest position, the width of the cut-open-side is 60.0 mm. We applied loads using the threaded rod until its width increased to a maximum of 61.5 mm. In 0.3 mm steps TSS was returned to its rest position and further compressed to 58.5 mm. All positions are kept for 30 s. The resulting calibration curve converts the voltage (mV) output of the Wheatstone bridge to Δ*L* (mm). The length change Δ*L* can be used to calculate ε according to equation (1). TSS responds to forces as low as 0.0098 N, which minimizes local tree part rigidification and makes it useable at all tree parts sampled in this study.

#### 6.2 Field measurements

On 29–30 June 2021 and 3–4 January 2022, field studies to test TSS under natural wind conditions were carried out at the forest research site Hartheim of the University Freiburg, located in the flat southern Upper Rhine Valley (47°56′04′’N, 7°36′02′’E, 201 m a.s.l.) At the time of the measurements, the Scots pine forest at the research site had a mean stand density of 550 trees per hectare and a mean stand height of 18.0 m [39].

**Fig. 6:**
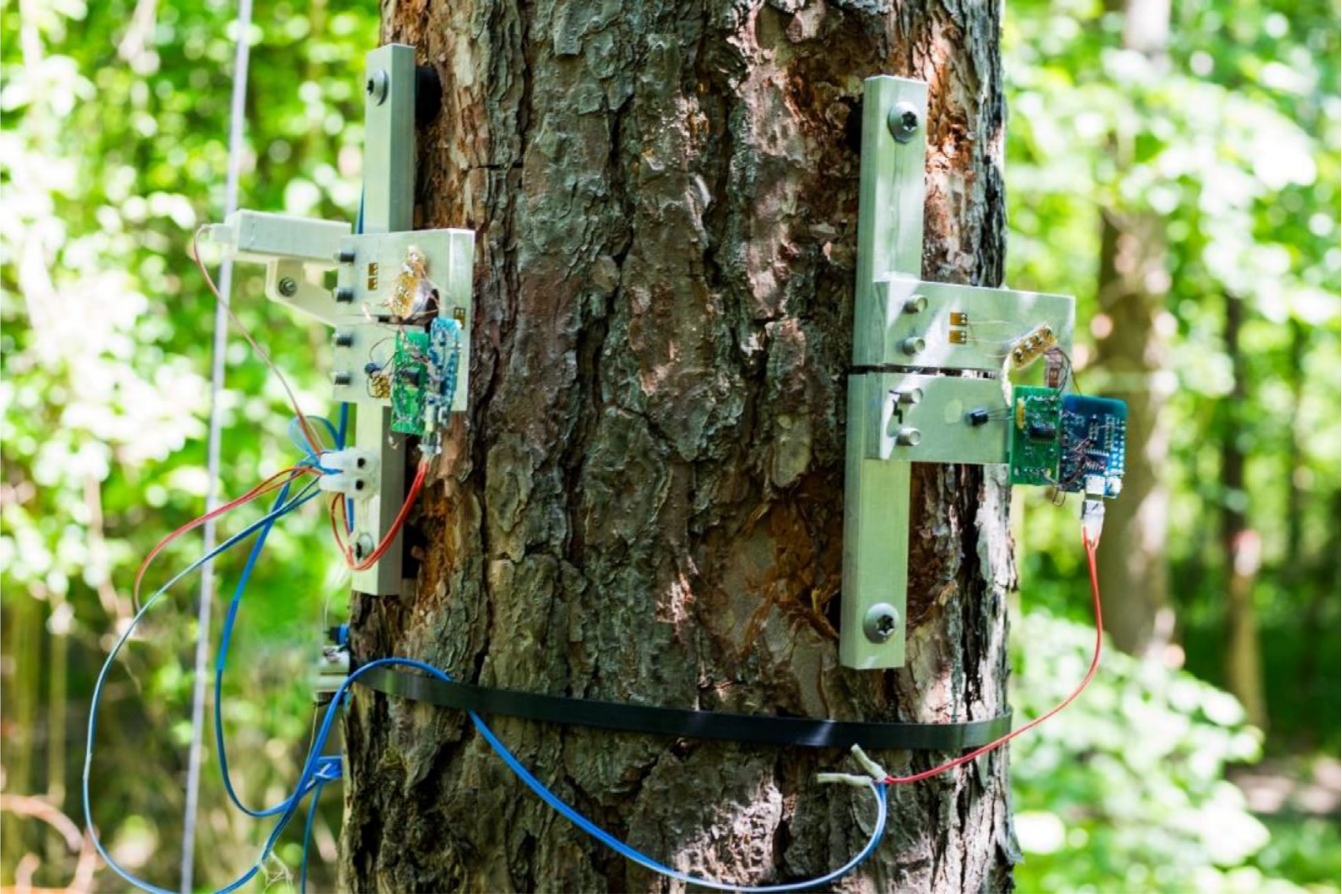
Two Tree Strain Sensors (TSS) mounted perpendicularly (z_2_ = 1/7H = 2.7 m) on a Scots pine (Pinus sylvestris L.) tree. On the left, a Tree Response Sensors (TRS) is installed next to the TSS.

To measure the above-ground response of the sample tree to wind loading, four TSS were mounted perpendicularly to an 18.6 m high Scots pine (*Pinus sylvestris* L.) tree at height *z_2_* = 1/7*H* and the stem base (*z_1_* = 0.2 m) to measure the stem strain in east–west (ε *_x_*) and north–south (ε *_y_*) directions (Fig. 6). The height *z_2_* = 1/7*H* height is assumed to represent the first antinodal point of vibration of a clamped-free beam and can be used to determine the wind-induced sway pattern [19], [40]. Additionally, one Tree Response Sensor (TRS) [33] each was installed at *z_1_* and *z_2_* to measure stem tilt (*T*) in east–west (*t_x_*) and north–south (*t_y_*) directions. The TRS measurements were used to compare the temporal dynamics of stem tilt and marginal wood fiber strain. Four TSS were mounted on the upper site of three exposed main roots 0.15–0.50 m away from the stem base to monitor the root response to wind loading.

The strain gauge extension arms were tightly screwed to the sampled tree parts at two points to prevent inaccurate measurements. To minimize strain gauge heating, we wrapped the sensors in space blankets.

The components of the 3D wind vector above the forest canopy were measured with an ultrasonic anemometer (type 81000VRE, R.M. Young Company, USA) installed on a 23 m scaffold tower. The horizontal distance between the ultrasonic measurement and sample tree was 6 m. The measured wind vector components were used to calculate the momentum flux (*M*), which is used to approximate the wind load acting on the sample tree [12].

#### 6.3 Tree pulling tests

Non-destructive static (SPT) and pull-and-release (PRPT) pulling tests validated TSS in the field under calm conditions. Two commercially available TreeQinetic (PTQ) elastometers (Argus electronic, Germany) were mounted next to the two TSS at the same height and directions as reference devices. They are typically used to measure strain of the stem during pulling tests, a standardized procedure to monitor the tree resistance against external loads [41]. The sample tree was subjected to two SPT and PRPT to validate TSS measurements. A 3-fold pulley (CT - Climbing Technology, Italia) combined with the PiCUS TreeQinetic system was used for the pulling tests. A pulling rope (Dyneema, Dynamica Ropes ApS, Denmark) was attached to the stem at 60 % of the tree height. The pulling force was increased until a stem base inclination of 0.25° was reached to prevent primary tree failure [42].

#### 6.4 Data processing

Outliers in the 10 Hz TSS data were removed using a Hampel filter. To remove drifts in the data, frequencies outside 0.003 to 2.5 Hz were attenuated using a bandpass filter. The same procedure was applied to the TreeQinetic elastometer data.

From the time series of directional strain measurements at the stem, ε *_x_* and ε *_y_*, the strain vector magnitude E was calculated:

(2) $$E=\sqrt{\varepsilon_{x}^{2}+\varepsilon_{y}^{2}}$$

On three primary roots (RT1-RT3), the strain was measured one-dimensionally along the longitudinal root axis (ε *_RT1_*, ε *_RT2_*, ε *_RT3_*). These measurements were carried out to study root response on the external loads acting on the above-ground tree parts. Two TSS were attached to northeast-facing RT1 (azimuth: 50°) at 15 cm (root diameter: 17.0 cm) and 50 cm (root diameter: 8.5 cm) distance from the stem base. One TSS was mounted to southwest-facing RT2 (root diameter: 5.3 cm; azimuth: 210°) and west-facing RT3 (root diameter: 5.5 cm; azimuth: 270°) at distances 43 and 38 cm from the base of the stem.

The time series of stem tilt components *t_x_* and *t_y_* were used to compute the tilt vector magnitude *T* [33]:

(3) $$T=\sqrt{t_{x}^{2}+t_{y}^{2}}$$

The fluctuations of the wind vector components *u* ($u^{\prime }$), *v* ($v^{\prime }$), and *w* ($w^{\prime }$) were the basis for the above-canopy momentum flux (*M*) calculation [43]:

(4) $$M\;=\;\sqrt{{\left(u^{\prime }w^{\prime }\right)}^{2}+{\left(v^{\prime }w^{\prime }\right)}^{2}}\;$$

To analyze and compare the frequency response of TRS and TSS, Welch power spectral density (*PSD*) estimates were calculated from the 10 Hz time series of *E* and *T*. The *PSD* calculation was performed over half-hourly intervals (18000 data points each). All spectra were normalized with their maximum to ensure their comparability.

#### 6.5 Spectral analysis

**Fig. 7:**
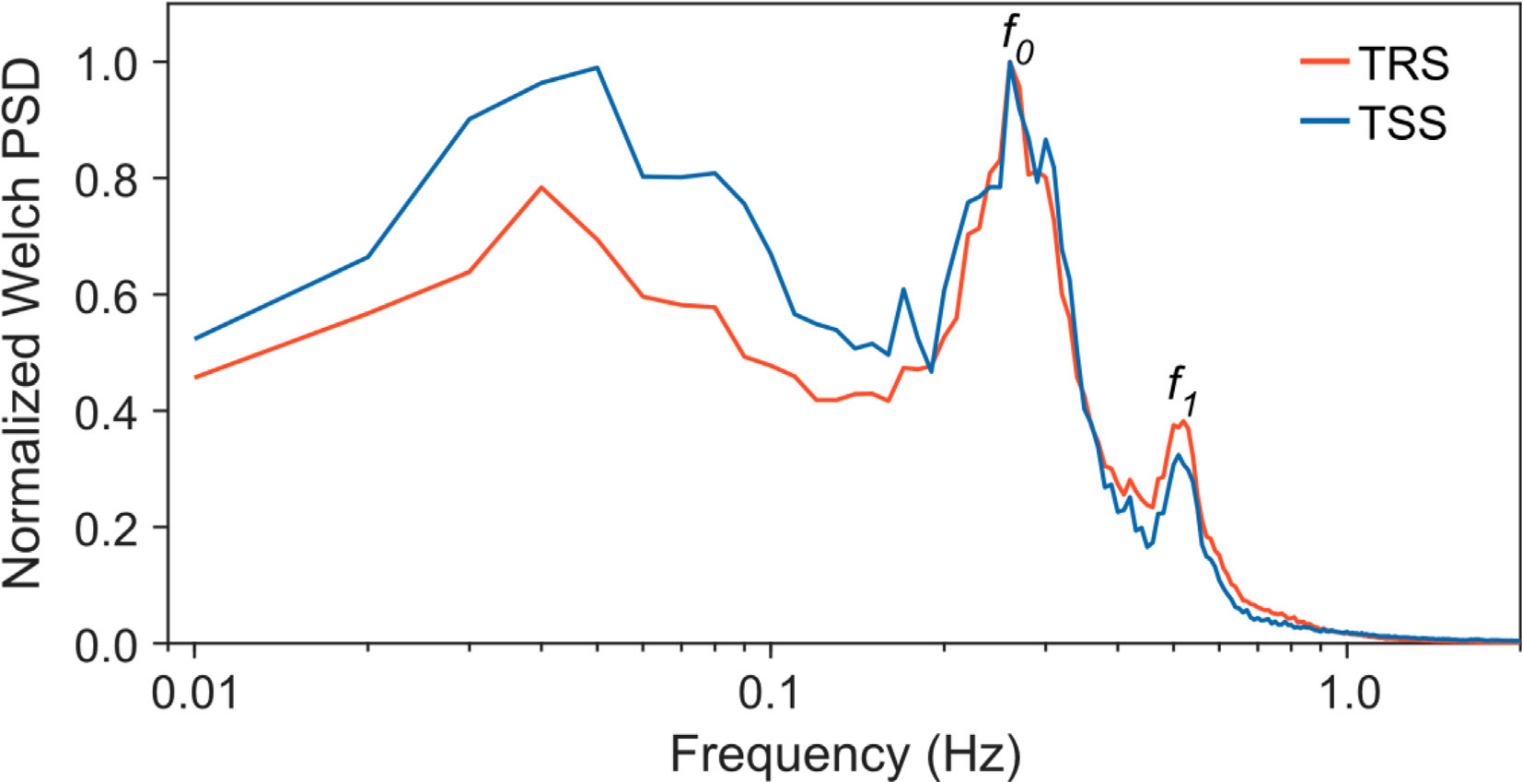
Mean normalized (by the maximum) Welch power spectral density (PSD) calculated over half-hourly intervals on 29–30 June 2021 using Tree Response Sensor (TRS) and Tree Strain Sensor (TSS) data (sampling rate 10 Hz), measured at height z_2_ = 1/7H = 2.7 m. The position of the fundamental sway frequency and the frequency representing the first harmonic is indicated by f_0_ and f_1_.

The mean normalized *E* and *T* Welch spectra are similar (Fig. 7), showing three local *PSD* maxima. The first maxima are caused by coherent turbulent structures in the above-canopy airflow passing the measurement site with 0.04 to 0.05 Hz frequencies. The second maximum is located in the range of the fundamental sway frequency (*f_0_* = 0.26). The third maximum indicates a harmonic (*f_1_* = 0.51) induced by the multimodal tree response to wind loading.

#### 6.6 Tree pulling test results

The measurement results of TSS and PTQ during the pulling tests are very similar, and the coefficient of determination (*R^2^*) between the analyzed data is at least 0.993. During all pulling tests, the *E* values measured with TSS and PTQ rise steeply with increasing force.

**Fig. 8:**
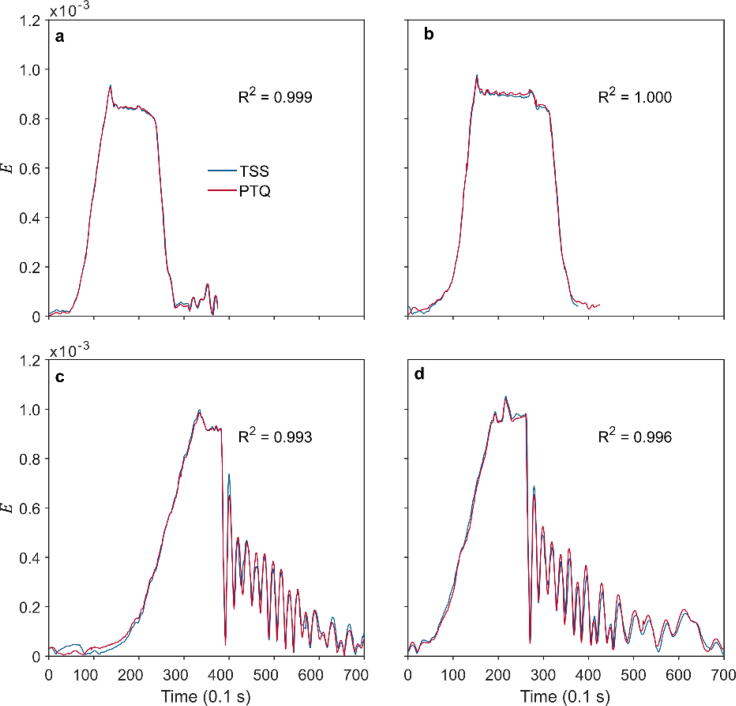
(a,b) Strain vector magnitude (E) calculated at height z_2_ = 1/7H = 2.7 m from Tree Response Sensors (TSS) and TreeQinetic elastometers (PTQ) measurements during two static pulling tests (SPT). (c,d) E available from TSS and PTQ measurements during two pull-and-release (PRPT) pulling tests. R^2^ is the coefficient of determination.

During the first static pulling test (Fig. 8a), they reach maximum values of 929 and 935 µE. Slightly higher *E* values at 965 and 976 µE were measured during the second static pulling test (Fig. 8b). After the maximum strain was recorded, the pulling force was gradually reduced until the sample tree returned to the rest position. The difference between the maxima of the TSS and PTQ measurements is less than 1 %. The mean absolute errors (*MAE*) associated with the TSS and PTQ measurements during the static pulling tests are *MAE =* 6 µE and *MAE* = 5 µE.

Similar good results were obtained for the two pull-and-release tests. The TSS and PTQ devices achieved a similar performance in the two tests shown in Fig. 8c and 8d. Like the static pulling tests, *E* measured with TSS and PTQ rises rapidly with increasing pulling force. After the *E* maximum was reached, the tree was released from the pulling rope to allow it to swing freely to its rest position. The decay of *E* is visible as an oscillating part of the represented time series. For the first pull-and-release test, *MAE* = -2 µE; for the second test *MAE* = 4 µE.

#### 6.7 Monitoring wind-induced tree response

**Fig. 9:**
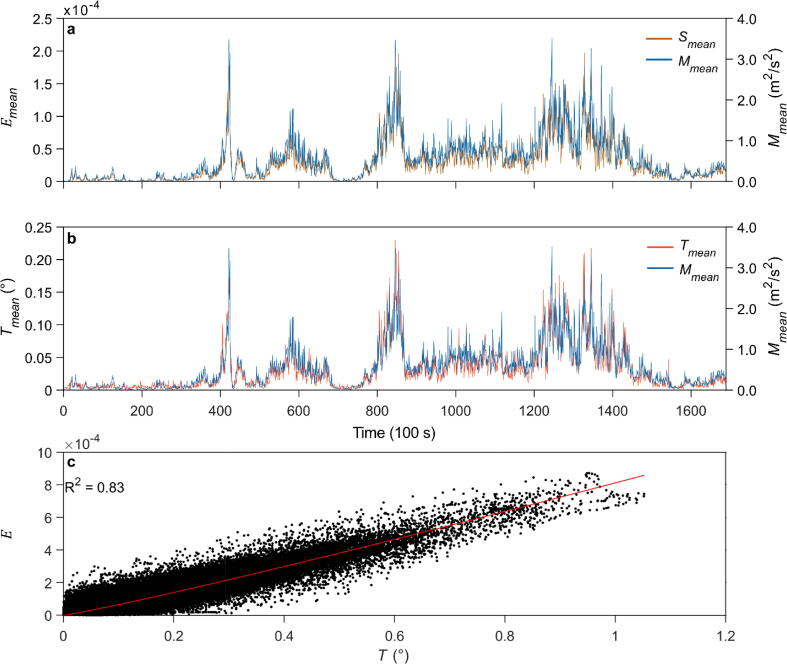
Time series (29–30 June 2021) of 100 s mean values of (a) strain (E) measured at height z_2_ = 1/7H = 2.7 m in the marginal fibers of the sample tree with the Tree Strain Sensor (TSS), and (b) stem tilt (T) measured with the Tree Response Sensor (TRS). The 100 s mean E and T values are plotted together with the above-canopy momentum flux (M) means values. (c) Relationship between E and T (sampling rate 10 Hz) at height z_2_ = 1/7H = 2.7 m.

Time series of 100 s mean (block average of 1000 samples) values of wind-induced *E* calculated at *z_2_* = 1/7*H* from TSS measurements are shown as an example for 29–30 June 2021 in Fig. 9a. The *E* mean values are presented with 100 s mean *M* values approximating the above-canopy wind load pattern. They are strongly correlated (*R^2^* = 0.88), and their correlation is slightly higher than the correlation of *T* measured with TRS and *M* (*R^2^* = 0.84) displayed in Fig. 9b. Based on this exemplary comparison of TSS and TRS, it can be concluded that TSS achieves at least similarly good measuring results as TRS, which has already been used in earlier studies [12], [33]. The linear relationship between *T* and *E* is displayed in Fig. 9c (sampling rate 10 Hz). It shows a tight correlation between *E* and *T,* with *R^2^* = 0.83.

**Fig. 10:**
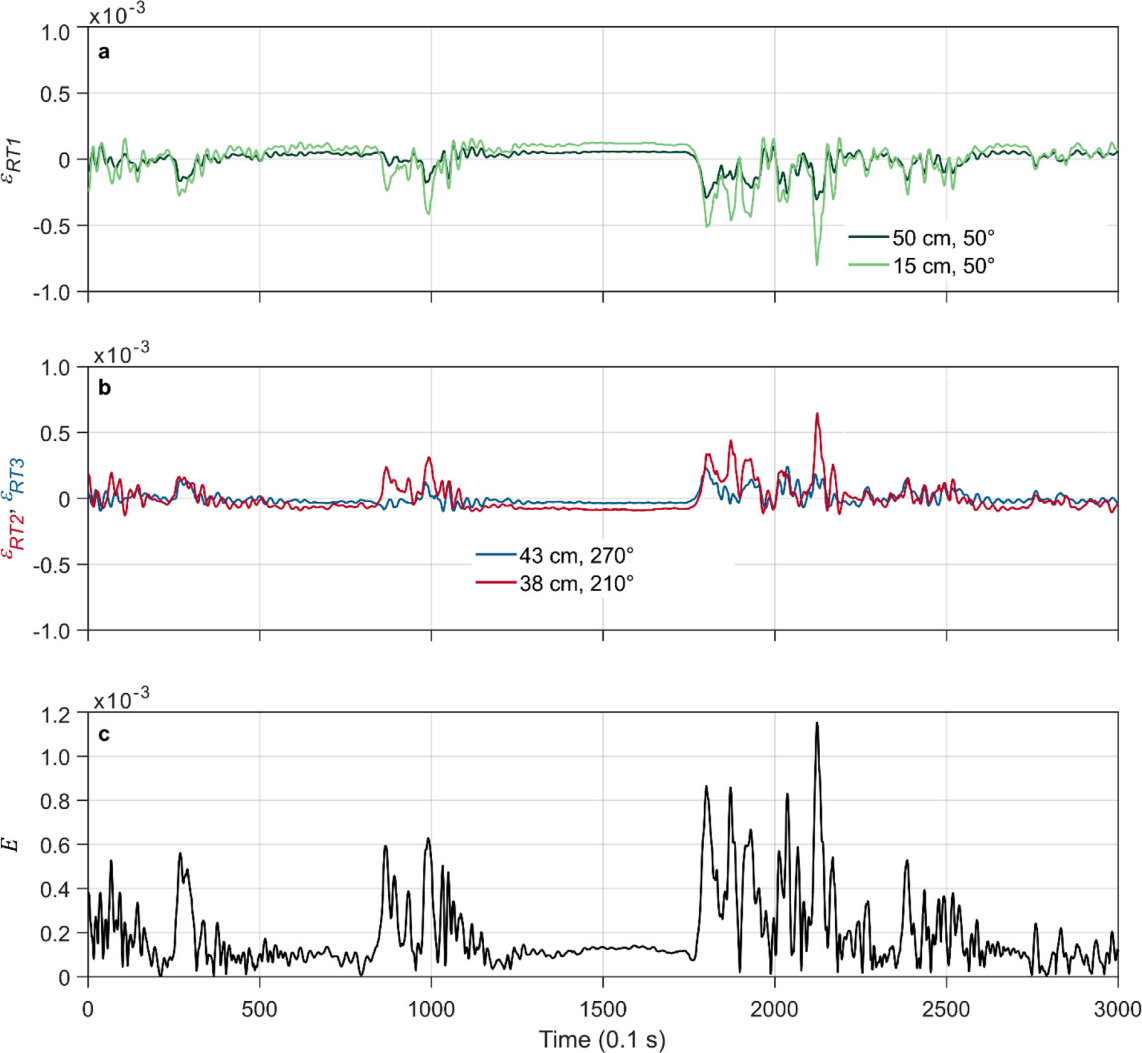
Five-minute sequence of 10 Hz TSS measurements (4 January 2022). (a) Strain along the longitudinal axis of the northeast-facing root RT1 (ε _Rt1_), measured at 15 cm and 50 cm distances from the stem base. Negative values indicate root elongation. (b) Strain measured along the longitudinal axis of southwest-facing root RT2 (ε _RT2_) and west-facing root RT3 (ε _RT3_) at distances 43 and 38 cm from the stem base. Positive values indicate root compression. (c) Strain vector magnitude (E) measured at the stem base (z_1_ = 0.2 m).

Fig. 10 presents 300 s sequences of measured root and stem strain (sampling rate 10 Hz). The time series shown for the two points along northeast-facing RT1 (growing in the main wind direction) are similar (*R^2^* = 0.87), with larger ε *_RT1_* measured closer to the stem base (Fig. 10a). The amplitudes of ε *_RT2_* measured at southwest-facing RT2 (growing against the main wind direction) match (*R^2^* = 0.97) the amplitudes of ε *_RT1_* but in the opposite direction. RT1 and RT2 growing at the opposite sides of the stem base show an antagonistic response pattern (Fig. 10b).

The response of RT3, oriented to the west, is less pronounced and weaker coupled with the response of RT1 (*R^2^* = 0.56) and RT2 (*R^2^* = 0.39). The response of RT1 and RT2 follows the strain pattern measured at the stem base (Fig. 10c)*.* Their reactions are more strongly correlated with *E* (*R^2^* = 0.75 and *R^2^* = 0.76) than the reactions of RT3 (*R^2^* = 0.42). The correlation between the response of the stem and the monitored roots indicates the dynamic transfer of elastic energy induced by wind loads acting on the aboveground tree parts to the roots.

### 7 Conclusions

The instructions summarized in this article allow the inexpensive Tree Strain Sensor TSS assembly. It reproduces the measuring results of a commercially available standard in the field. The use of the full-bridge configuration yields better results than the previously used half-bridge configurations. It is possible to precisely measure the response of tree parts to external loads such as pulling tests and natural wind loading. The straightforward integration of TSS into TreeMMoSys allows easy, wireless, and synchronized data collection from multiple TRS and TSS with one system and increases the range of TreeMMoSys applications. The integrated system makes it possible to track the vibration behavior of tree parts down to the ground. Its widespread use helps gather crucial information to understand better wind-tree interaction and the formation of storm damage in forests.

## Declaration of Competing Interest

The authors declare that they have no known competing financial interests or personal relationships that could have appeared to influence the work reported in this paper.

## Figures and Tables

**Table:** 

ID	Part	Source
01-02		[32]
03-04	Aluminum parts	Local Workshop
05	WEMOS D1 mini	https://www.ebay.de/itm/275126072440?chn=ps&var=575400657862&_trkparms=ispr%3D1&amdata=enc%3A1JwTmuwB2SQa2sh_gRDYHxQ89&norover=1&mkevt=1&mkrid=707-134425-41852-0&mkcid=2&itemid=575400657862_275126072440&targetid=1596011700870&device=c&mktype=pla&googleloc=9042403&poi=&campaignid=14472331666&mkgroupid=125370287694&rlsatarget=pla-1596011700870&abcId=9300652&merchantid=112143330&gclid=CjwKCAjwuYWSBhByEiwAKd_n_hx62T79ovCejIspIkmNaflu2bO6ITooSW8liE6rfhv93MOpmdhWOhoCynwQAvD_BwE
06	HX711 board	https://www.komputer.de/zen/index.php?main_page=product_info&products_id=475&zenid=s0omnelrc81tq0v205hpqjo1r6
07	Screw M6 × 20 mm	https://schrauben-expert.de/Linsenkopfschraube-ISO-7380-1-M6-x-20-Edelstahl-A2
08	Woodscrews, dimensions tbd	For example https://www.bauhaus.info/holzschrauben-spanplattenschrauben/spax-universalschraube/p/10019503?gclid=CjwKCAjwuYWSBhByEiwAKd_n_jTiuHe4OMMLeLpyMyyQ9KmS9N4PwyXSSlHSTABTqVlhAp5rqzBv3xoCLHgQAvD_BwE&ef_id=CjwKCAjwuYWSBhByEiwAKd_n_jTiuHe4OMMLeLpyMyyQ9KmS9N4PwyXSSlHSTABTqVlhAp5rqzBv3xoCLHgQAvD_BwE:G:s&s_kwcid=AL!5677!3!402866511583!!!g!875375476964!&cid=PSEGoo8400533753_85436250189&pla_campid=8400533753&pla_adgrid=85436250189&pla_prpaid=875375476964&pla_prid=10019503&pla_adt=pla&pla_prch=online&pla_stco=
09	3M Scotch-Weld® SF20 Instant Adhesive	https://www.klebeband.eu/3m-scotch-weld-sw-sf-20-klar-20g-cyanacrylat-klebstoff
10	Enamelled wire, 0.35 mm	https://www.conrad.de/de/p/beli-beco-kupferlackdraht-aussen-durchmesser-inkl-isolierlack-0-33-mm-10-m-1437332.html
11	Mibenco liquid rubber	https://www.amazon.de/mibenco-72829010-Flüssiggummi-Weiß-Matt/dp/B00TTMKFV4/ref=sr_1_4?__mk_de_DE=ÅMÅŽÕÑ&keywords=flüssiggummi+weiß+mibenco&qid=1569489504&sr=8-4
12	Double Sided Foam Adhesive Tape	https://www.ebay.de/itm/393946119250?chn=ps&_trkparms=ispr%3D1&amdata=enc%3A1w8R5rVZsSCisjC4rc8hKjw5&norover=1&mkevt=1&mkrid=707-134425-41852-0&mkcid=2&itemid=393946119250&targetid=1596011700910&device=c&mktype=pla&googleloc=9042403&poi=&campaignid=14472331666&mkgroupid=125370287694&rlsatarget=pla-1596011700910&abcId=9300652&merchantid=494540045&gclid=CjwKCAjwuYWSBhByEiwAKd_n_mYMFcRwGGMkYmAm2m3U-8UUDNLLwQXL_sfT8BSIin1Drs5qV-bRJBoC2GMQAvD_BwE
13	Rescue Blanket	https://www.amazon.de/dp/B00ICWM66O?tag=shopping-de-amazon-suggestedresults-1-21&linkCode=osi&th=1&psc=1
14	Taidacent BF350-3AA pre-soldered strain gauges	https://de.aliexpress.com/item/1005003778935667.html?gatewayAdapt=glo2deu&_randl_currency=EUR&_randl_shipto=DE&src=google&src=google&albch=shopping&acnt=494-037-6276&slnk=&plac=&mtctp=&albbt=Google_7_shopping&albagn=888888&isSmbAutoCall=false&needSmbHouyi=false&albcp=12554800262&albag=127990761348&trgt=1284054470089&crea=de1005003778935667&netw=u&device=c&albpg=1284054470089&albpd=de1005003778935667&gclid=CjwKCAjwuYWSBhByEiwAKd_n_mySiUpQOTer--k5eCscuINPeiM4yCtPzoWxQGtAbsikjc8or4UuCRoCjCsQAvD_BwE&gclsrc=aw.ds&aff_fcid=2cb477d90cc640af98f8b7f474ee90e8-1648473162741-03085-UneMJZVf&aff_fsk=UneMJZVf&aff_platform=aaf&sk=UneMJZVf&aff_trace_key=2cb477d90cc640af98f8b7f474ee90e8-1648473162741-03085-UneMJZVf&terminal_id=341bdca7c3474aa495a435d84dec371d&afSmartRedirect=y
15	tesafilm® eco & clear tesafilm® self-adhesive tape	https://www.bueroshop24.de/tesa-eco-clear-klebefilm-transparent-15-0-mm-x-10-0-m-3-rollen-774174?srpId=5b3742a2db684071300276728143552f&storeType=B2C&lkz=1159483&obt=11&kwdid=5440413105-15911154461-129630311182-pla-335177835751&gclid=CjwKCAjwuYWSBhByEiwAKd_n_kGx8EGueR3dEQEIUqYkgLFthkzIs3E9KDbBfOYSoRqo6V5wFJzdzhoCPeAQAvD_BwE
16	Circuit board	https://www.pollin.de/p/punktrasterplatine-440458
17	Twin-stranded-wire 0.14 mm^2^	https://www.pollin.de/p/zwillingslitze-2x-0-14-mm2-rot-schwarz-10-m-561804?utm_source=google&utm_medium=fshopping&gclid=CjwKCAjwuYWSBhByEiwAKd_n_lmHzwD3zJIEwViEDB86dC0DLd4FCQe6vKKGeWMEqaKD61T-h6A38BoChooQAvD_BwE
18	Micro USB Plug Type B	https://www.reichelt.de/micro-usb-stecker-typ-b-5-polig-usb-micro-st-p124013.html?PROVID=2788
19	Conformal Coating M.G. Chemicals 419D	https://de.rs-online.com/web/p/schutzlacke/1462871
